# *Notes from the Field*: Enteropathogenic *Escherichia coli* Outbreak at a Child Care Center — Oregon, August 2021

**DOI:** 10.15585/mmwr.mm7114a3

**Published:** 2022-04-08

**Authors:** Kimberly E. Bonner, McKenna Carter, Christopher Zielinski, Karim Morey, Lillian McLitus, Emilio DeBess, Julie Hatch, Richard Leman

**Affiliations:** ^1^Epidemic Intelligence Service, CDC; ^2^Public Health Division, Oregon Health Authority; ^3^Hood River County Health Department, Hood River, Oregon; ^4^Oregon State Public Health Laboratory, Hillsboro, Oregon.

On August 16, 2021, the Oregon Health Authority Public Health Division (OPHD) was notified of two pediatric cases of Shiga toxigenic *Escherichia coli* among members of the same household. Each of the patients received a positive polymerase chain reaction test result for Shiga toxin in a stool specimen. *E. coli* O157:H7 was subsequently isolated from stool culture from both patients. During routine case investigation, the local health department determined that one patient, aged 2 years, had attended an in-home child care center. OPHD visited the child care center on August 18 to conduct case ascertainment among staff members and children, share recommendations for rapid isolation and exclusion of those ill, observe infection prevention practices during diaper changing, and educate staff members on infection prevention measures for toys and high-touch surfaces. The investigation team requested parental consent and attempted to collect clinical information on gastrointestinal symptoms during July 30–August 18 and stool specimens from all staff members and children on the day of the visit. A child care center staff member followed up with other staff members and children who were not present on the day of the visit to obtain clinical information and provide them with specimen collection kits and instructions. Stool specimens were placed in Cary-Blair transport medium, transported to OPHD, and tested for 22 enteric pathogens using the BioFire FilmArray Gastrointestinal Panel (BioFire Diagnostics, LLC).

Clinical information was provided for each of the 17 children and four staff members enrolled or employed at the child care center. Among these, six of 17 (35%) children and one of four staff members reported diarrhea, vomiting, or other gastrointestinal symptoms. Stool specimens were collected from 18 (86%) children and staff members. Initially, culture for* E. coli *O157 was performed on 10 specimens; all were negative for *E. coli *O157:H7. Twelve specimens were acceptable for testing using BioFire; nine specimens contained evidence of an enteric pathogen, including seven with enteropathogenic *E. coli* (EPEC), four with norovirus, and one each with rotavirus, sapovirus, astrovirus, and *Campylobacter*. Four specimens yielded more than one pathogen. Among seven persons with EPEC, three were symptomatic, as were three of four with norovirus infection. Two persons infected with EPEC were coinfected with norovirus, one of whom was symptomatic. After reviewing laboratory results, the local health officer recommended temporarily closing the child care center for 7 days; the child care center complied, household members of children and staff members were informed of the outbreak and asked to monitor for symptoms, and no additional cases were reported.

This outbreak during August 2021 is the first EPEC outbreak detected in Oregon. Several patients experienced coinfection with other enteric pathogens. EPEC causes diarrhea by adhering to the small intestine endothelium, damaging microvilli, and affecting absorption (*1*). The public health implications of asymptomatic EPEC infection remain unclear (*2*). With the advent of multiplex gastrointestinal assays, in use in Oregon since 2015, more EPEC outbreaks are likely to be detected (*3*). During 1971–2018, 58 EPEC outbreaks were reported to the National Outbreak Reporting System; 43 (74%) of these outbreaks were detected during 2016–2018. Among the 13 EPEC outbreaks at child care centers, 12 were detected during 2016–2018.* As detection of EPEC increases through broader use of multiplex assays, a need exists to develop guidance for case and outbreak management of EPEC outbreaks in congregate settings and child care centers.

## Footnotes

* The National Outbreak Reporting System (NORS) provides a publicly available download of all NORS Dashboard data, including etiology, exposure location, and year of report. https://wwwn.cdc.gov/norsdashboard/
